# Thianthrene polymers as 4 V-class organic mediators for redox targeting reaction with LiMn_2_O_4_ in flow batteries

**DOI:** 10.1038/s41598-023-32506-7

**Published:** 2023-04-07

**Authors:** Kan Hatakeyama-Sato, Karin Sadakuni, Kan Kitagawa, Kenichi Oyaizu

**Affiliations:** 1 Department of Applied Chemistry, Waseda University, Tokyo, 169-8555 Japan; 2 Advanced Research and Innovation Center, DENSO CORPORATION, Aichi, 470-0111 Japan

**Keywords:** Polymer chemistry, Batteries

## Abstract

Redox targeting reaction is an emerging idea for boosting the energy density of redox-flow batteries: mobile redox mediators transport electrical charges in the cells, whereas large-density electrode-active materials are fixed in tanks. This study reports 4 V-class organic polymer mediators using thianthrene derivatives as redox units. The higher potentials than conventional organic mediators (up to 3.8 V) enable charging LiMn_2_O_4_ as an inorganic cathode offering a large theoretical volumetric capacity of 500 Ah/L. Soluble or nanoparticle polymer design is beneficial for suppressing crossover reactions (ca. 3% after 300 h), simultaneously contributing to mediation reactions. The successful mediation cycles observed by repeated charging/discharging steps indicate the future capability of designing particle-based redox targeting systems with porous separators, benefiting from higher energy density and lower cost.

## Introduction

Redox-flow battery is a crucial technology for storing renewable energy with scalability and reasonable cost^1–5^. The cells typically have two individual tanks containing electrolytes and redox-active molecules, displaying reversible charging and discharging reactions even over 10^3^ cycles^4^. Their simple and easy-to-maintenance configuration is preferred to store electricity on a much larger scale than conventional lithium-ion batteries.

As active materials, vanadium oxides are used for commercial flow batteries^6^. Recently, organic-based molecules, such as 2,2,6,6-tetramethylpiperidine 1-oxyl (TEMPO), anthraquinone, sulfur, and viologen, are actively studied as alternative materials^1–4,7–9^. Their abundant resource and flexible molecular tunability could afford cheaper, safer, and higher-performance flow batteries.

One critical drawback of the traditional cell design can be the much smaller volumetric energy density than regular lithium-ion batteries. Due to the limited solubility or dispersibility of the molecules, their typical molar concentration ranges from 0.1 to 2 mol/L, corresponding to the catholyte or anolyte capacities of 3 to 50 Ah/L^2–4,6^. In contrast, lithium-ion batteries easily exceed the concentration of 10 mol/L (270 Ah/L) because of the high densities of inorganic materials (> 3 g/cm^3^)^10–16^.

**Figure 1:**
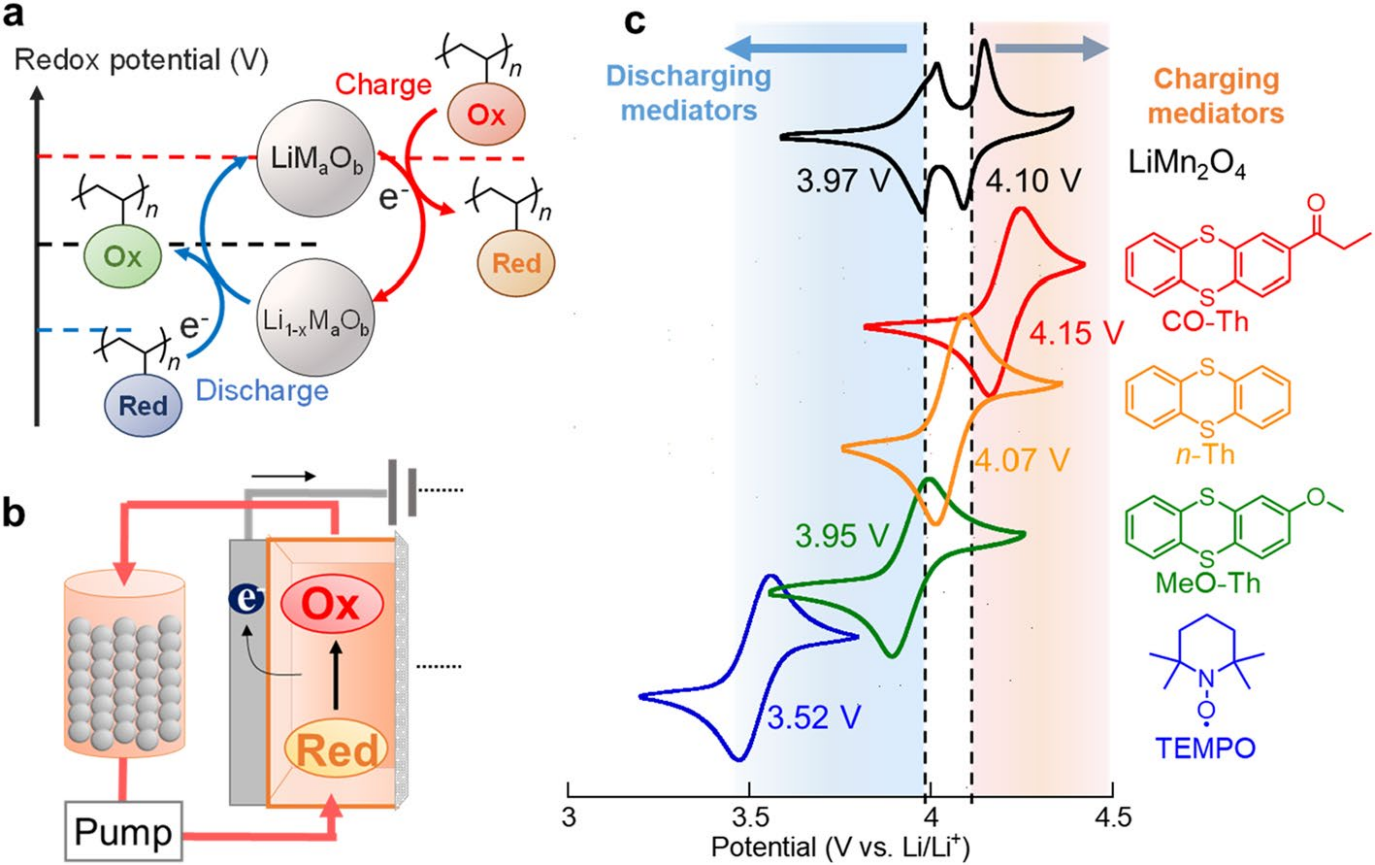
(**a**) Scheme of redox targeting reaction of lithium metal oxides using redox-active polymer mediators. (**b**) Illustration of a catholyte tank filled with densely packed cathode-active materials. (**c**) Cyclic voltammograms of LiMn_2_O_4_ (scanned at 0.2 mV/s) and various redox mediators (5 mM, scanned at 10 mV/s) measured in 1 M Lithium bis(trifluoromethanesulfonyl)imide (LiTFSI) in ethylene carbonate/diethyl carbonate. The observed currents were normalized for comparison.

A concept of redox targeting reaction, or redox mediation, was proposed to solve the energy density problem of flow systems^10–13,15,16^. Inorganic active materials are packed in the tanks, and redox-active organic compounds flow so that the molecules can electrochemically oxidize or reduce the inorganic compounds. The charge transfer occurs according to the potential differences between inorganic materials and mediators. Its fast heterogeneous reaction could afford the charging/discharging rate of over 10 C for inorganic materials^13^. Therefore, the reacted mediators can transport charges throughout the cell (Fig. 1a,b).

The redox targeting reaction occurs with combinations of metal oxides and redox mediators^16^. There are variations of the mediators, represented by organic redox-active molecules, metallocene, ferricyanide, iodine, and polysulfides^15,16^. As high-density materials, inorganic metal oxides have mainly been examined, in addition to solid-state or gel-like organic compounds^17–19^. Initially, a prototype system was demonstrated using metallocene mediators and LiFePO_4_ and TiO_2_ as metal oxides, yielding a capacity of over 240 Ah/L per tank^13^. The value was around five times larger than conventional vanadium flow cells. The cell maintained about 70% against the initial capacity after ca. 50 cycles, which did not reach the average level of flow cells (> 1000)^4^, but showed the possibility for practical applications.

A remaining challenge of redox targeting systems is increasing the voltage. The potentials of the standard materials, LiFePO_4_ and TiO_2_, are 3.4 and 1.8 V vs. Li/Li^+^, respectively^13,16^. The expected voltage of the cell would be only 1.6 V, which is much smaller than conventional lithium-ion batteries (> 3 V). The low voltage comes from the few candidates of redox mediators working at higher (> > 3.4 V) or lower (< < 1.8 V) potentials, whereas conventional inorganic materials offer wide variations (e.g., graphite: 0.1 V, LiCoO_2_: 3.7 V, and LiMn_2_O_4_: around 4 V)^7,20,21^.

Reported potentials of most redox-active molecules have been limited up to 3.8 V^7,20,21^. The values were less than 3 V for most quinones^22^, 3.4 V for ferrocene^13^, 3.6 V for TEMPO^4^, around 3.7 V for 2,2,5,5-tetramethylpyrrolidine 1-oxyl (PROXYL)^21^, and ca. 3.7 V for triphenylamine derivatives^23,24^. Introduction of electron-withdrawing groups (e.g., SO_2_) to the redox units could boost potentials over 4 V, whereas stability is sacrificed as a trade-off^25^. Aromatic heterocycles, such as thianthrene (4.1 V)^26,27^, phenoxazine (3.7 V)^28^, and fluoflavin (3.7 V)^20,29^, are relatively new-class redox units showing high potentials. Still, they have not joined the mainstream of active materials for flow systems, certainly because of poorer solubility than the aliphatic species (i.e., due to $\pi - \pi$ stacking) and insufficient data for cyclability^20,26–29^.

In this report, we tackled the high potential region (> 4 V) of organic mediators for hybrid flow systems and employed LiMn_2_O_4_ as the inorganic material. LiMn_2_O_4_ shows a redox capacity of ca. 120 mAh/g and a high density of 4.2 g/cm^3^, yielding an excellent theoretical capacity of 500 Ah/L. For mediated charging of the material, thianthrene-based organic mediators were designed with different molecular geometry (i.e., small molecules, dissolved polymers, and nanoparticles). We found large molecular radii of the polymeric materials were beneficial for suppressing unfavorable crossover reactions through separators, even porous ones. Although the oxidized states of thianthrenes were not highly stable, they could be used in flow cells because of the mediation design; the oxidized cations were rapidly reduced to normal states by reacting with the inorganic material. The findings will lead to higher energy density and longer life flow cells with organic/inorganic hybrid design.

## Results

### Examination of thianthrene and TEMPO derivatives as redox mediators

We focus on LiMn_2_O_4_ as a target cathode-active material for mediation. The compound exhibited reversible redox reactions at 3.97 and 4.10 V vs. Li/Li^+^ in a conventional lithium-ion electrolyte (1 M LiTFSI in ethylene carbonate/diethyl carbonate, Fig. 1c). Compared to most traditional cathode materials (LiCoO_2_ and its derivatives), LiMn_2_O_4,_ offer flatter plateaus during charge/discharge reactions owing to the stable spinel structure^30,31^. The flatness is favored for faster mediation because redox targeting reactions proceed by the potential differences between metal oxides and redox mediators^16^. Manganese is also cheaper and more resource-abundant than metals used in conventional lithium-ion batteries (e.g., cobalt and nickel).

An oxidation mediator to LiMn_2_O_4_ should have a potential over 4 V. However, few organic compounds exhibit reversible redox reactions in the region because degradation often occurs by reacting with electrolytes or by themselves^20^. This article examined thianthrene derivatives, offering redox at over 4 V^26,27,32–34^. Several derivatives were synthesized: methoxy- (MeO-Th), normal (*n*-Th), and ethylcarbonyl-thianthrene (CO-Th). The methoxy and carbonyl groups were introduced as electron-donating and -withdrawing groups, respectively.

**Scheme 1:**
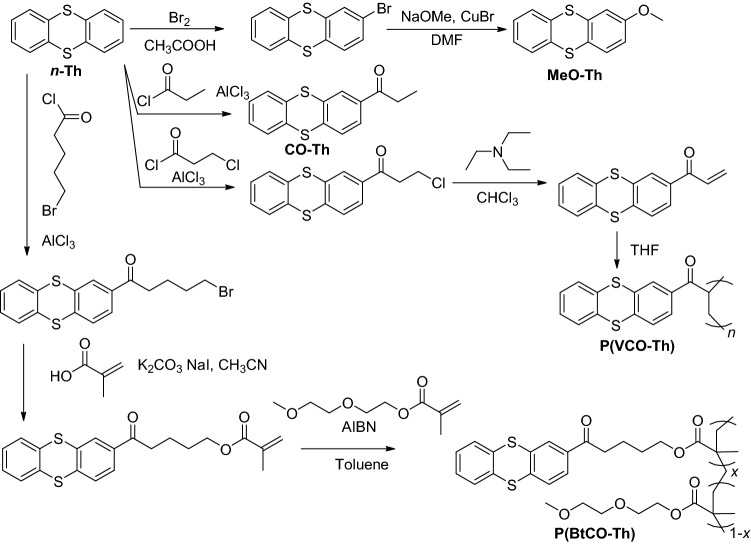
Synthesis of thianthrene derivatives.

The thianthrene compounds exhibited reversible redox reactions at around 4 V (MeO-Th: 3.95 V, *n*-Th: 4.07 V, and CO-Th: 4.15 V, Fig. 1c, Scheme 1, Scheme S1, Scheme S2, Figure S1, Figure S2). The potentials of the compounds sandwiched LiMn_2_O_4_: MeO-Th would mainly work as a reducing mediator and *n*-Th as an oxidant. We also examined TEMPO, a robust redox-active molecule with organic batteries^35^. The compound showed a potential of 3.52 V, available as a discharging mediator to LiMn_2_O_4_ (Fig. 1c).

H-type cells were fabricated to examine the redox-targeting reactions. We prepared cells with four different catholyte conditions: Entry (1) disperse only LiMn_2_O_4_, (2) add extra TEMPO and *n*-Th as discharging and charging mediators, (3) extra MeO-Th and CO-Th, and (4) extra TEMPO and CO-Th (Table 1). Entry 1 is a control condition without mediation. In entry 2, LiMn_2_O_4_ will be oxidized by *n*-Th at around 4.1 V and reduced by TEMPO at around 3.5 V. In entry 3, both charging (4.15 V) and discharging (3.95 V) potentials will increase by the use of higher potential thianthrene mediators, CO-Th and MeO-Th, respectively. Only discharging voltage will decrease with the use of TEMPO mediator in entry 4.

**Table 1:** Charge/discharge results for the H-type cells.

Entry	Mediator (for discharging/charging)	LiMn_2_O_4_ (eq)	Capacity (mAh/L)			Coulombic efficiency (%)
			Charge	Discharge	Theoretical	
1	None	10	2	1	670	78
2	TEMPO/Th	10	557	370	804	67
3	MeO-Th/CO-Th	10	502	110	804	22
4	TEMPO/CO-Th	10	671	519	804	77
5	PTMA/P(BtCO-Th)	0	130	102	134	79
6		10	384	279	804	73
7	PTMA/P(VCO-Th)	0	107	73	134	68
8		10	250	173	804	69

**Figure 2:**
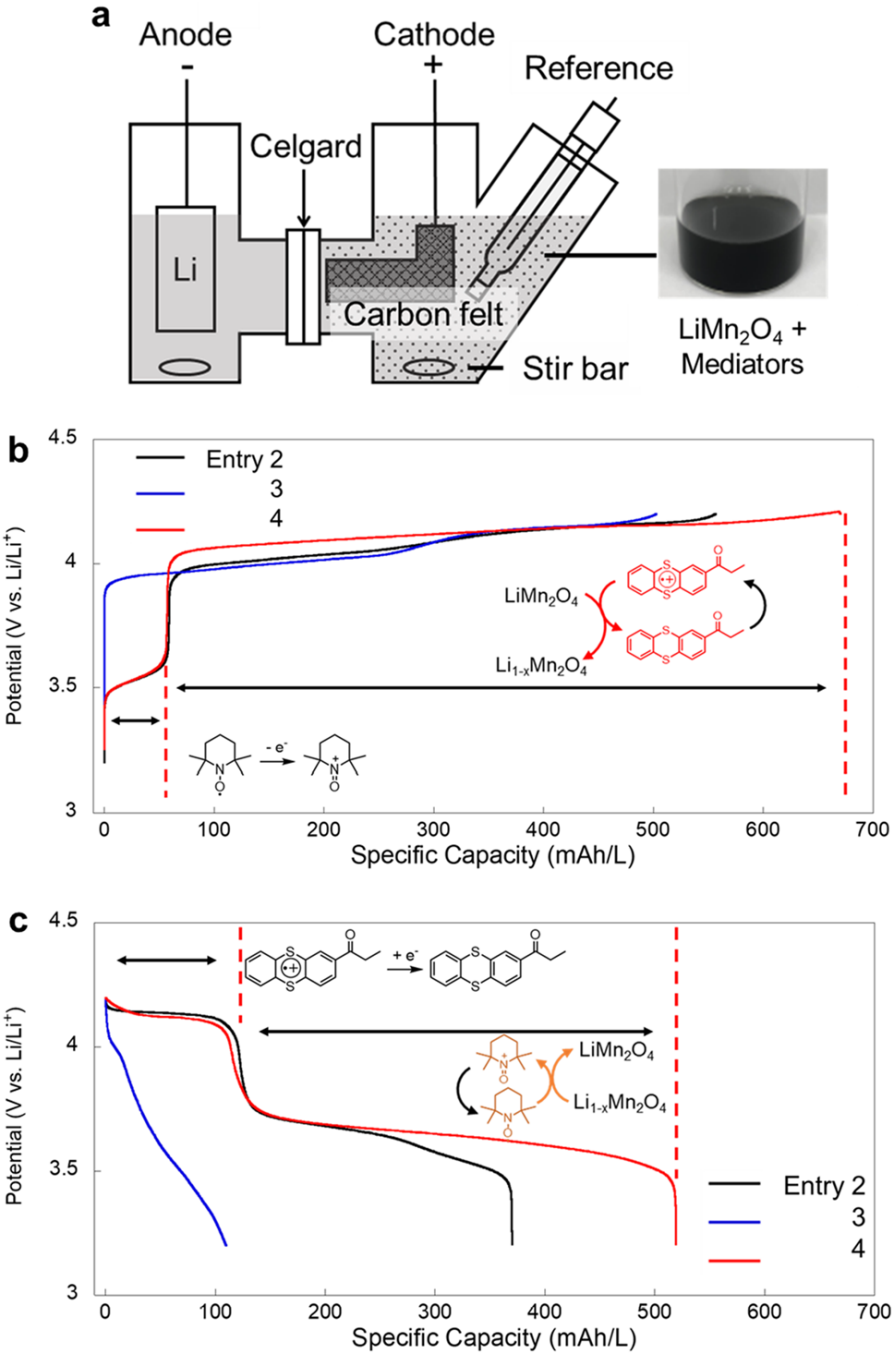
(**a**) Configuration of an H-type cell. In the catholyte, LiMn_2_O_4_ was dispersed at a concentration of 670 mAh/L. Charging and discharging mediators were dissolved at a concentration of 67 + 67 mAh/L. (**b**) Charging and (**c**) discharging curves for the cells (Entries 2–4). The operation rate was set to be 0.5 C against the mediators.

The experimental setup is shown in Fig. 2a. Carbon felt was used as a current collector, and lithium foil was introduced as the counter electrode. The counter cell was separated by a conventional separator (Celgard 2400, pore size ca. 40 nm). The charging/discharging capacity was set to be 0.5 C against the mediators. The catholyte was stirred vigorously to disperse inorganic oxides and mix them with mediators. The theoretical capacities of LiMn_2_O_4_ and each mediator were adjusted as 670 and 67 mAh/L, respectively. The values were much smaller than practical flow cells and redox targeting devices (> > 1000 mAh/L)^1,16^. However, the simple configuration of the cells was favored to examine the fundamental kinetics of the mediation reactions.

A control cell of only LiMn_2_O_4_ (Entry 1) displayed almost zero capacity during charging and discharging reactions (Table 1, curves are not shown). The inorganic particles had no opportunity to contact a current collector for redox reactions^27^. On the other hand, the addition of mediators increased the charging capacity significantly (entries 2–4, Fig. 2b,c). The obtained values exceeded 500 mAh/L, which could be explained only by the redox targeting reactions because the mediators' capacity was only 67 + 67 = 134 mAh/L.

When TEMPO and *n*-Th were introduced as mediators (Entry 2), two charging plateaus at ca. 3.6 and 4.1 V were observed. The charging capacity at 3.6 V was around 70 mA/h, indicating that TEMPO was oxidized without mediation. The second plateau showed a capacity of about 500 mAh/L. Here, *n*-Th was repeatedly charged and chemically oxidized LiMn_2_O_4_ through redox targeting reactions (Fig. 1a).

The first plateau curve was almost identical even when the charging mediator was changed to CO-Th. The similar response was caused because TEMPO did not affect the charging mediation (Entry 4). On the other hand, the total charging capacity increased from about 560 to 670 mAh/L. The difference was attributed to the higher redox potential of CO-Th (4.15 V) than LiMn_2_O_4_ (3.97 and 4.10 V), necessary to extract charge from LiMn_2_O_4_ at higher potentials.

From the viewpoint of cell voltage, the most intelligent design could be using MeO-Th and CO-Th as discharging and charging mediators, respectively (Entry 3). The experimental voltage exceeded 4 V. However, the cell exhibited the smallest charging capacity of 502 mAh/L of the mediation conditions.

After charging the cells, the catholytes were discharged at the same rate of 0.5 C. In contrast to charging, more significant differences were observed. The smallest capacity of 110 mAh/L and Coulombic efficiency of only 22% were detected by the MeO-Th/CO-Th mediators (Entry 3). Further, we observed almost no plateau in the discharging curve. The unexpectedly small charging and discharging capacities were certainly caused by the unfavorable degradation of charged thianthrenes and incomplete mediation; side reactions with electrolytes were reported^26,32–34^.

When TEMPO, a more stable mediator, was introduced for discharging (Entry 2, 4), much larger capacities were obtained. For the TEMPO and *n*-Th couple (Entry 2), the discharging capacity and Coulombic efficiencies were 370 mAh/L and 67%, respectively. The values became further larger with TEMPO/CO-Th (519 mAh/L and 77%) because CO-Th had a higher charging potential than *n*-Th and showed faster mediation with LiMn_2_O_4_.

The increase of Coulombic efficiency by TEMPO indicated an unexpected advantage of the redox targeting design. Unfortunately, the oxidized thianthrenes were not highly stable and thus the compound was not on the mainstream of organic active materials^26,32–34^. Still, the instability drawback could be partially solved with redox mediation because the unstable oxidized species could return to the stable normal state after passing the charge to inorganic materials. If the mediation proceeds fast, the degradation could become negligible. Although the current low Coulombic efficiency (70%) indicates the necessity of more optimized molecular and device design, the redox targeting system gives new chances to less stable redox-active compounds.

### Synthesis of redox-active polymer mediators

**Figure 3:**
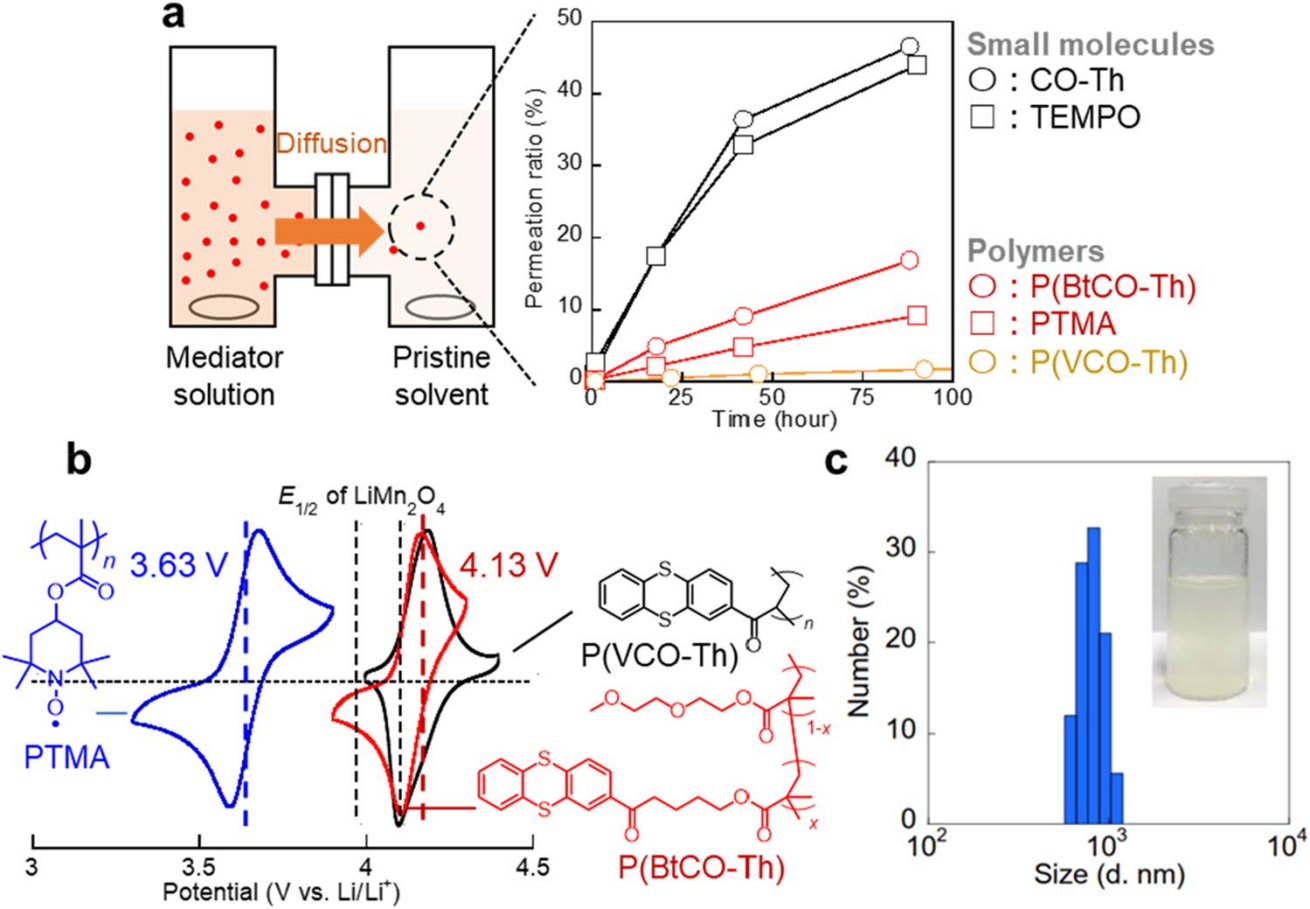
(**a**) Configuration of the cell for permeation test and related experimental result. The mediators were dissolved or dispersed in the carbonate solvent and kept stirred to proceed with permeation to the other cell with only solvents. UV–Vis measurements estimated the permeation amount. (**b**) Cyclic voltammograms of the three polymers. Soluble PTMA and P(BtCO-Th) were dissolved at a concentration of 5 mM and scanned at 50 mV/s. A carbon composite electrode was prepared for insoluble P(VCO-Th) and scanned at 50 mV/s. c, Dynamic light scattering spectrum for P(VCO-Th) dispersed at a concentration of 1 mM in the carbonate solvent.

A drawback of the examined molecular mediators is quick permeability through battery separators. When TEMPO and CO-Th solutions were separated by a conventional separator (Celgard 2400), most molecules diffused to the opposite cell in some days (Fig. 3a). Non-porous electrolyte membranes are often introduced to avoid permeation^4^, but their higher cost and lower conductivity are not always optimal for practical applications.

An alternative solution to avoid crossover is enlarging redox mediators. Permeation can be suppressed by introducing polymer or oligomer structures; their larger hydrodynamic radii will suppress permeation^2^. Introduction of crosslinked polymer particles is an ultimate solution: if the radii of the rigid particles are larger than pore sizes, the crossover will not occur unless the particles are mechanically broken^36,37^.

We synthesized several thianthrene and TEMPO polymers (Fig. 3b, Figure S3-Figure S5, Scheme S3-Scheme S6). Poly(vinyl ketone-substituted thianthrene) (P(VCO-Th)) was a polymerized form of CO-Th. A new polymer, poly(butyl ketone-substituted thianthrene) (P(BtCO-Th)), was synthesized as another oxidizing mediator. The latter was obtained as a copolymer with diethylene glycol monomethyl ether methacrylate (DMEM) to improve solubility (Table S1). A TEMPO-substituted polymer, poly(TEMPO-substituted methacrylate) (PTMA), was prepared via radical polymerization as a soluble compound in the electrolyte. The radical polymer displayed reversible redox at *E*_1/2_ = 3.63 V.

A thianthrene polymer P(VCO-Th) had a large molecular weight of *M*_w_ = 9.5 × 10^4^ (*M*_w_/*M*_n_ = 1.5) and was insoluble in the carbonate electrolyte. On the other hand, the polymer behaved as dispersible nanoparticles in the electrolyte after processing by an ultrasonic homogenizer (mean radius of around 800 nm, Fig. 3c). The compound exhibited reversible redox at 4.13 V, which was almost identical to CO-Th.

Soluble thianthrene polymer P(BtCO-Th) was synthesized after tuning the copolymerization ratio with the ethylene glycol units (DMEM, Table S1). When the initial ratio of monomer and DMEM was set as 1/1 (mol/mol), the experimental copolymerization ratio became 2/1 because of the slightly different polymerization reactivities of the monomers. The product was not soluble in the carbonate electrolyte. When the monomer ratio was set as 1/2, the actual proportion of the thianthrene and ethylene glycol units became 1/1. The higher amount of DMEM made the polymer soluble in the electrolyte. The polymer exhibited reversible redox reactions at 4.13 V.

### Electrochemical characteristics of polymer mediators for redox targeting cells

The electrochemical kinetics of the three polymers were studied using rotating disk electrode (RDE) measurements under diluted conditions of 2 mM for redox sites (Figure S6-Figure S9). The apparent diffusion coefficient for charge transfer *D*_app_ and standard electrode reaction constant *k*^0^ were estimated from Koutecký-Levich plots (Table 2).

**Table 2:** Electrochemical kinetic parameters for the thianthrene and TEMPO derivatives estimated by RDE.

Compound	Type	*D*_app_ (×10^–6^ cm^2^/s)	*k*_0_ (×10^–3^ cm/s)
CO-Th	Soluble molecule	2.7	3.5
TEMPO		3.3	5.0
P(VCO-Th)	Polymer particle	0.027	0.097
P(BtCO-Th)	Soluble polymer	0.17	0.27
PTMA		0.19	0.32

Small molecules displayed typical kinetic constants: *D*_app_ = 2.7 $\times$ 10^–6^ cm^2^/s and *k*_0_ = 3.5 $\times$ 10^–3^ cm/s for CO-Th and *D*_app_ = 3.3 $\times$ 10^–6^ cm^2^/s and *k*_0_ = 5.0 $\times$ 10^–3^ cm/s for TEMPO. The values were comparable to the reported values of small molecules^2^. Soluble polymers gave about 1/10 times smaller values: *D*_app_ = 0.17 $\times$ 10^–6^ cm^2^/s and *k*_0_ = 0.27 $\times$ 10^–3^ cm/s for P(BtCO-Th) and *D*_app_ = 0.19 $\times$ 10^–6^ cm^2^/s and *k*_0_ = 0.32 $\times$ 10^–3^ cm/s for PTMA. The decreases were attributed to the larger hydrodynamic radii and slower physical diffusion^36^. Polymer nanoparticles, P(VCO-Th), displayed the smallest values of *D*_app_ = 0.027 $\times$ 10^–6^ cm^2^/s and *k*_0_ = 0.097 $\times$ 10^–3^ cm/s., which can also be explained by the largest hydrodynamic radii (ca. 800 nm)^36^. Although the polymerization decreased the rate constants, the products were applicable to electrochemical reactions for redox cells (vide infra).

As a benefit of macromolecules, their larger radii impeded the unfavorable permeation through the porous separator (Fig. 3a). The permeated amount of P(VCO-Th) nanoparticles was only 3% after 300 h. The value will become almost zero after complete purification of the polymer (i.e., exclude smaller molecules by comprehensive dialysis). Even dissolved polymers displayed about 1/4 times slower permeation than the control monomers. Synthesis of larger molecular weight polymers (*M*_w_ > 10^5^) would almost perfectly suppress the crossover due to the larger hydrodynamic radii than pores, which is an ongoing topic of our reserch^38^.

Finally, we fabricated H-type cells using the synthesized polymer. Four types of the cells were examined: Entry (5) soluble PTMA/P(BtCO-Th) pair, (6) Entry 5 + LiMn_2_O_4_, (7) PTMA and insoluble P(VCO-Th) pair, and (8) Entry 7 + LiMn_2_O_4_ (Table 1). Entries 5 and 7 were control experiments to evaluate redox reactions of polymers without inorganic materials. Entries 6 and 8 were designed to proceed with redox targeting reactions. In both conditions, PTMA worked as a soluble discharging mediator. P(BtCO-Th) or P(VCO-Th) was introduced as soluble or insoluble charging mediators in entries 6 or 8, respectively. The operation rate was set to be 0.25 C, which was half of the small molecule systems; the slower diffusion of the polymers required lower rates.

**Figure 4:**
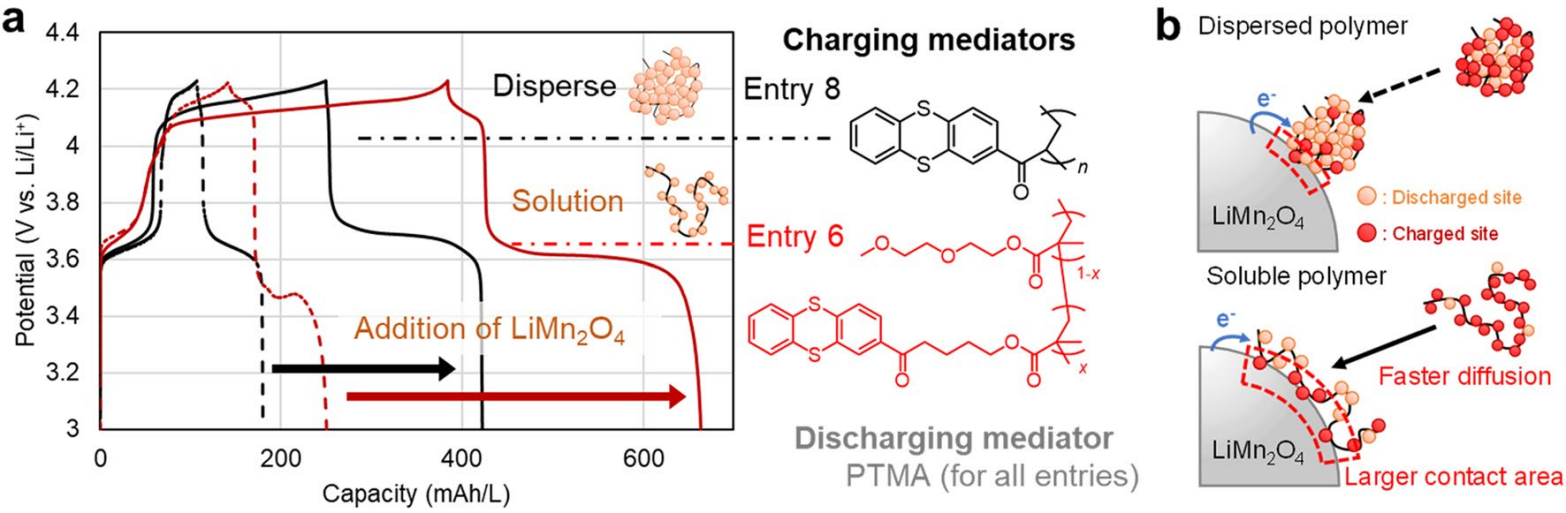
(**a**) Charge/discharge curves for the LiMn_2_O_4_ and polymer mediator cells, operated at 0.25 C for mediators (Entries 5–8). Dashed (Entries 5, 7) and solid (Entries 6, 8) lines represent the results without and with the existence of LiMn_2_O_4_. (**b**) Comparison of dispersed and soluble redox mediators to oxidize the inorganic material.

The soluble pair of PTMA/P(BtCO-Th) (Entry 5) displayed a charging capacity of 130 mAh/L and Coulombic efficiency of 79% (Fig. 4a). The experimental capacity was comparable to the theoretical one (134 mAh/L). Even larger nanoparticles of P(VCO-Th) offered a charging capacity of 107 mAh/L (Entry 7), indicating that the slower electrochemical kinetics of the polymers were not significantly problematic.

The experimental capacities increased significantly by adding LiMn_2_O_4_ for redox targeting reactions (Entries 6, 8). In the case of soluble PTMA/P(BtCO-Th) mediators, the charging capacities and Coulombic efficiency reached 384 mAh/L and 73%, respectively (Entry 8). The achieved charging capacities by the polymer mediators were not so high as the small molecule system (TEMPO/CO-Th: 671 mAh/L, Entry 4). The difference was caused by the slower diffusion of the polymers and less frequent collision against LiMn_2_O_4_ to induce medication. Detailed electrochemical kinetics must be studied in the future for more optimal molecular design.

An unexpected result was that even P(VCO-Th) nanoparticles were beneficial as mediators. Because of the slower diffusion, the particle–particle collision should be drastically slower than solution systems (Fig. 4b, Entry 8). However, the experimental values of 250 mAh/L and 69% for the nanoparticle system indicated that the particle collisions induced charge mediations. Although larger molecular radii result in lower diffusivity and less frequency to collide inorganic materials, the design offer potential advantages for future flow cell design of particle systems: high energy density (> 1 mol/L concentration of mediators)^36^, simple porous separator without crossover, and intrinsically low cost. Tackling the kinetic limit by a new molecular design would enable highly efficient cells.

Another challenge of the hybrid cells is increasing cycle performances. When the polymer cells were charged and discharged repeatedly, the experimental capacities decreased gradually (Fig. S10). The charging/discharging was almost reduced by half with the hybrid cells of P(BtCO-Th) and P(VCO-Th) (Entries 6 and 8) after five cycles. The main reason could be the instability of the oxidized thianthrenes; the species showed less than 100 cycle durability in normal battery form^26^. Further cell optimizations, such as carbon coating on LiMn_2_O_4_ and faster flow, are needed to accelerate charge mediation and to return the unstable oxidized thianthrenes to normal states, yielding longer cycle life.

## Conclusions

In conclusion, we examined prototype cells for redox-targeting flow batteries with higher energy density. The introduction of 4 V-class inorganic materials, such as LiMn_2_O_4_ (around 4 V vs. Li/Li^+^), has been a challenge for the redox targeting system. This work demonstrated that thianthrene derivatives and their polymers could catalytically oxidize LiMn_2_O_4_. The insufficient stability of the charged thianthrenes is a problem for long-life batteries, but it could be alleviated by faster charge mediation with the inorganic oxide. Even polymer nanoparticles can induce redox-targeting reactions via solid-to-solid electron transfer, offering an opportunity of introducing inexpensive porous separators in flow cells. Revealing the detailed electrochemical kinetics of the polymer mediators with metal oxides and enhancing cycle performances by improving molecular design are ongoing issues of our continuous study.

## Methods

### Materials

LiMn_2_O_4_ was purchased from FUJIFILM Wako Chemicals Co. (practical capacity of 110 mAh/g. Lithium bis(trifluoromethanesulfonyl)imide (LiTFSI) was purchased from Kanto Chemical Co. Carbonate solvents (ethylene carbonate/diethyl carbonate = 3/7 in volume) were obtained from Kishida Chemical Co. TEMPO, thianthrene, propionyl chloride, 5-bromovaleryl chloride, methacrylic acid, and diethylene glycol monomethyl ether methacrylate (DMEM) were obtained from TCI chemicals Co. Other reagents and solvents were purchased from Kanto Chemical Co., TCI chemicals Co., FUJIFILM Wako Chemicals Co., or Sigma-Aldrich. All compounds were used as received unless noted otherwise.

### Synthesis of 2-methoxythianthrene (MeO-Th) (Scheme S1)

Thianthrene (4.0 g, 18.5 mmol) and 60 mL of acetic acid were placed in a 300 mL three-necked flask. After purging by nitrogen, bromine (6.81 g, 42.6 mmol) dissolved in 20 mL of acetic acid was added dropwise by cannulation. After heating at 80 °C for 6 h, the mixture was cooled to room temperature and quenched with the aqueous sodium of thiosulfate (6.81 g/100 mL). The product was extracted with chloroform, washed with brine), and subjected to silica gel column chromatography (using hexane as eluent, *R*_f_ = 0.34). Recrystallization with hexane gave 2-bromotianthrene as a white solid (FAB-MS: m/z = 295.92 (calcd), 205.77 (found), 60% yield).

2-Bromotianthrene (588 mg, 2.00 mmol), 5 M sodium methoxide in methanol (0.8 mL, 4.00 mmol), and 1 mL *N*,*N*-dimethylformamide (DMF) were placed in a 50 mL Erlenmeyer flask. Copper(I) bromide (28.6 mg, 0.200 mmol) was added, and after nitrogen replacement, the reaction was continued at 120 °C for another 2 h. The product was extracted with chloroform and washed with water. Then, it was purified using silica gel column chromatography (chloroform/hexane = 1/3, *R*_f_ = 0.29). MeO-Th was obtained as a white solid after drying under reduced pressure (FAB-MS : m/z = 246.02 (calcd), 245.92 (found), Figure S1, 30% yield).

### Synthesis of 1-(thianthren-2-yl)propan-1-one (CO-Th) (Scheme S2)

Aluminum chloride (1.66 g, 12.5 mmol) was dispersed in 20 mL of dichloromethane in a 100 mL Erlenmeyer flask, and thianthrene (3.0 g, 13.9 mmol) was added slowly. Propionyl chloride (1.09 mL, 12.5 mmol) dissolved in 10 mL of dichloromethane was added slowly dropwise while cooling to 0 °C. After nitrogen replacement, the reaction was stirred at 40 °C for 18 h at reflux. After the reaction, the solution was slowly poured into ice water with hydrochloric acid (10 mmol). After the extraction with dichloromethane and washing with brine, the crude was purified via silica gel column chromatography (chloroform/hexane = 1/1, *R*_f_ = 0.42), and dried under reduced pressure. CO-Th was obtained as a pale yellow solid (FAB-MS: m/z = 272.03 (calcd), 272.01 (found), Figure S2, 27% yield).

### Synthesis of poly(vinyl ketone-substituted thianthrene) (P(VCO-Th)) (Scheme S3)

The polymer was prepared according to our previous procedure with modification^27^. Aluminum chloride (1.85 g, 13.9 mmol) was dispersed in 18 mL of dichloromethane in a 300 mL three-necked flask. While cooling to 0 °C, 3-chloropropionyl chloride (1.07 mL, 11.1 mmol) was added slowly. After nitrogen replacement, thianthrene (3.0 g, 13.9 mmol) dissolved in 45 mL of dichloromethane was added dropwise by cannulation, and the reaction was stirred at 40 °C for 18 h at reflux. The solution was slowly poured into ice water, extracted with dichloromethane and washed with brine. Then, it was subjected to silica gel column chromatography (chloroform/hexane = 2/1, *R*_f_ = 0.58), and dried under reduced pressure to afford 3-chloropropionic acid thianthrene **1** as a yellow liquid (FAB-MS : m/z = 305.99 (calcd), 306.97 (found), 32% yield).

The precursor **1** (1.25 g, 4.08 mmol) was dissolved in 10 mL of chloroform in a 50 mL Erlenmeyer flask, and after nitrogen replacement, triethylamine (1.14 mL, 8.16 mmol). The mixture was stirred at room temperature for 16 h. The mixture was washed with 0.1 M hydrochloric acid (twice), NaHCO_3_ aqueous solution (twice), and brine. The crude was subjected to silica gel column chromatography (chloroform/hexane = 2/1, *R*_f_ = 0.42). The product was dried under reduced pressure to give thianthrene-substituted vinyl ketone (**2**) as a yellow solid (FAB-MS : m/z = 270.02 (calcd), 271.00 (found)). The vinylketone monomer was highly reactive and difficult to be recrystallized. Polymerization would start as a dissolved solution at room temperature (88% yield).

For polymerization, **2** (0.50 g) was dissolved in 25 mL of THF in a 100 mL flask and heated at 65 °C for 3 h after nitrogen replacement. No radical initiator was needed to start polymerization because the monomer polymerized spontaneously. After the reaction, the solution was concentrated by evaporation, precipitated and purified in methanol, centrifuged, and dried under reduced pressure to afford P(VCO-Th) as a pale yellow powder (65% yield, Figure S3). The molecular weight was calculated by gel permeation chromatography (GPC) in THF (*M*_w_ = 9.5 × 10^4^, *M*_w_/*M*_n_ = 1.5, polystyrene standard). P(VCO-Th) was soluble in chloroform and THF, but insoluble in the carbonate electrolyte. After homogenizing the solution by a probe sonicator　(Sonifier, BRANSON), P(VCO-Th) was stably dispersed in the electrolyte. Dynamic light scattering estimated the particle size to be about 800 nm.

### Synthesis of poly(butyl ketone-substituted thianthrene) (P(BtCO-Th)) (Scheme S4, Scheme S5)

Aluminum chloride (3.08 g, 23.1 mmol) and 10 mL of dichloromethane were dispersed in a 300 mL three-necked flask. Then, thianthrene (5.00 g, 23.1 mmol) dissolved in 60 mL of dichloromethane was added dropwise. After cooling to 0 °C, 5-bromovalenyl chloride (3.05 mL, 23.1 mmol) dissolved in 10 mL of dichloromethane was added slowly dropwise, and the reaction was refluxed at 40 °C for 24 h after nitrogen replacement. The solution was slowly poured into 150 mL of ice water, extracted with dichloromethane (50 mL × 3) and washed with brine (150 mL × 3). It was subjected to silica gel column chromatography (chloroform/hexane = 1/1, *R*_f_ = 0.29), and recrystallized with hexane to afford the pale yellow crystal as a monomer precursor (**3**) (28% yield, FAB-MS : m/z = 377.97 (calcd), 377.66 (found)).

For monomer synthesis, potassium carbonate (1.64 g, 11.9 mmol), methacrylic acid (0.600 mL, 7.12 mmol), and 4-methoxyphenol (3 cups with a spatula) as polymerization inhibitor were added to 40 mL of acetonitrile in a 100 mL Erlenmeyer flask under an argon atmosphere. After stirring for 10 min, **3** (1.50 g, 3.95 mmol) and sodium iodide (60 mg, 0.40 mmol) as a catalyst were added, and the mixture was refluxed at 80 °C for 48 h. The reaction mixture was then evaporated by evaporation. The reaction solution was reduced to half solvent by evaporation, poured into 100 mL of pure water, aliquoted (extraction with dichloromethane, washing with 0.1 M sodium hydroxide solution (150 mL × 2) and brine (150 mL)), and subjected to silica gel column chromatography (ethyl acetate/hexane = 1/9, *R*_f_ = 0.30) and dried under reduced pressure to afford methacrylate monomer (**4**) as a yellow viscous liquid (67% yield, FAB-MS: m/z = 384.09 (calcd), 383.84 (found), Figure S4). The compound could be easily polymerized and needed to be cooled for storage.

Just before polymerization, polymerization initiators were removed by the following procedure. Monomer **4** or diethylene glycol monomethyl ether methacrylate (DMEM) was dissolved in 3 mL of toluene. Then, 3 mL of 1 M NaOH solution was added and stirred for 5 min. Then, the water layer was removed. The NaOH addition and removal steps were repeated. Next, 3 mL of brine was added and stirred for 5 min, and the aqueous layer was removed. The remaining water was removed by magnesium sulfate.

For polymerization, **4**, DMEM, and 5 mol% AIBN (2,2′-azodiisobutyronitrile) as an initiator were dissolved in toluene at a molar ratio of 1/1 (Run 1) or 1/2 (Run 2), respectively. In the case of Run 2, **4** (192 mg), DMEM (188 mg), and AIBN (12.3 mg) were dissolved in 5 mL toluene. Radical polymerization was carried out at 65 °C for 5 h. P(BtCO-Th) was obtained as a pale yellow solid after precipitation purification into hexane. The copolymerization ratio *x* was calculated to be 0.66 (Run 1) and 0.49 (Run 2) from the results of ^1^H-NMR. The polymer with low content of ethylene glycol moieties (Run 1) was insoluble in the electrolyte. A soluble polymer was obtained with Run 2 (Figure S5, Run 1: 65% yield and Run 2: 52% yield).

### Synthesis of poly(TEMPO-substituted methacrylate) (PTMA) (Scheme S6)

PTMA was synthesized according to a previous report of reversible addition − fragmentation chain-transfer (RAFT) polymerization^39^. 2,2,6,6-Tetramethyl-4-piperidyl methacrylate (1.80 g, 8.00 mmol), and cumyl dithiobenzoate (27.2 mg, 0.100 mmol), and AIBN (3.3 mg, 0.020 mmol) were dissolved in 5 mL toluene in a 30 mL flask. The mixture was degassed by freeze–pump–thaw (FPT) cycling (Vacuum 3 min, Ar flow 2 min × 5 times). Radical polymerization was conducted by heating the solution at 75 °C for 18 h. After quenching by cooling the mixture, it was precipitated into cooled hexane with ice water. After drying under reduced pressure, the PTMA precursor with RAFT adduct (RAFT-PTMA′) was obtained as a pink powder (yield: 1.47 g).

RAFT-PTMA′ was subsequently reacted with an excess amount of AIBN to remove the RAFT agent. RAFT-PTMA′ (500 mg), AIBN (170 mg), and 9 mL of toluene were added to a 50 mL flask, and degassed by FPT (three times). The solution was stirred at 75 °C for 18 h. PTMA precursor (PTMA′) was obtained as a pale pink powder (350 mg yield).

For oxidation, PTMA′ (300 mg, 1.33 mmol) was dissolved in 6 mL of dichloromethane in a 30 mL flask, cooled to 0 °C, and a solution of *m*CBPA (740 mg, 3.99 mmol) in dichloromethane (6 mL) was added dropwise by cannulation. After stirring at room temperature for 3 h, 40 mL of dichloromethane was added, followed by washing with 50 mL of sodium carbonate aqueous solution (pH = 13, three times). The solution was precipitated into hexane, and dried under reduced pressure to afford PTMA as a light pink powder (0.20 g yield). The radical concentration was estimated to be 79% (88 mAh/g) by SQUID measurement. The molecular weight was estimated to be *M*_w_ = 1.71 × 10^4^ (*M*_w_/*M*_n_ = 1.11) by GPC. The product was soluble in the carbonate electrolyte.

### Electrochemical measurements

Electrochemical measurements were conducted with a three-electrode system using 1 M LiTFSI in ethylene carbonate/diethyl carbonate (3/7 in volume) as the electrolyte. A conventional potentiostat (BAS Inc. ALS 660D) was employed for measurements. During cyclic voltammetry and RDE measurements, a glassy carbon disk (diameter of 3 mm) was selected as a working electrode. A platinum coil and an Ag/AgNO_3_ electrode were employed as the counter and reference electrodes. The observed potentials were converted to the value against Li/Li^+^ by measuring the compounds with lithium reference electrodes under the same condition. Cyclic voltammograms of LiMn_2_O_4_ and P(VCO-Th) were recorded as carbon composite electrodes (active material/vapor-grown carbon fiber/poly vinylidene difluoride = 1/8/1 in weight) coated on glassy carbon substrates.

Charging/discharging measurements were conducted using H-type cells. In two cells, 4 mL of the electrolyte was added for each. The cells were separated by a porous separator (Celgard 2400). A mediator (2.5 mM in redox site concentration) and LiMn2O4 (670 mAh/L) were added to the catholyte side. A carbon felt was introduced as a current collector. A reference electrode (Ag/AgNO_3_) was inserted into the working side. A lithium foil was used in the counter. The cells were stirred vigorously to disperse LiMn_2_O_4_ during measurements. Operation rates were 0.5 C for small molecular mediators and 0.25 C for polymer mediators.

### Permeation measurement

The mediators were dissolved in an ethylene carbonate/diethyl carbonate (3/7 in volume) solvent at 2.5 mM. The solution was put on one side of an H-type cell, and a blank solvent was added to the opposite side (3 mL for each). The cells were connected through a porous separator (Celgard 2400) and stirred for several days to observe permeation. The permeated amounts were estimated by the light absorbance during UV–Vis measurement. Standard solutions of thianthrene or TEMPO were employed for calibration. The permeation ratio was defined so that the initial state corresponded to 0% and the complete permeation to 50%.

## Supplementary Information

Supplementary Information.
