# Supplementary material for: Thianthrene polymers as 4 V-class organic mediators for redox targeting reaction with LiMn2O4 in flow batteries
Source: Sci Rep. 2023 Apr 7;13:5711. doi: 10.1038/s41598-023-32506-7 (PMC10082199; doi:10.1038/s41598-023-32506-7)
Supplement: Supplementary file 1 — Supplementary Information. [file 41598_2023_32506_MOESM1_ESM.docx]

# Supplementary Information

**Thianthrene polymers as 4 V-class organic mediators for redox targeting reaction with LiMn_2_O_4_ in flow batteries**

Kan Hatakeyama-Sato,^1*^ Karin Sadakuni,^1^ Kan Kitagawa,^2^ Kenichi Oyaizu^1*^

1 Department of Applied Chemistry, Waseda University, Tokyo 169-8555, Japan
2 Advanced Research and Innovation Center, DENSO CORPORATION, Aichi 470-0111, Japan
*satokan@toki.waseda.jp (K.H.) / oyaizu@waseda.jp (K.O.)

Supplementary Figures and Schemes


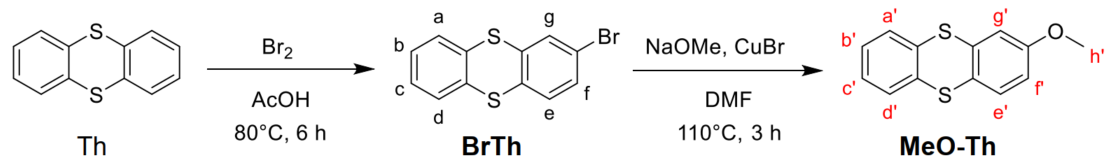


Scheme S1. Synthesis of MeO-Th.


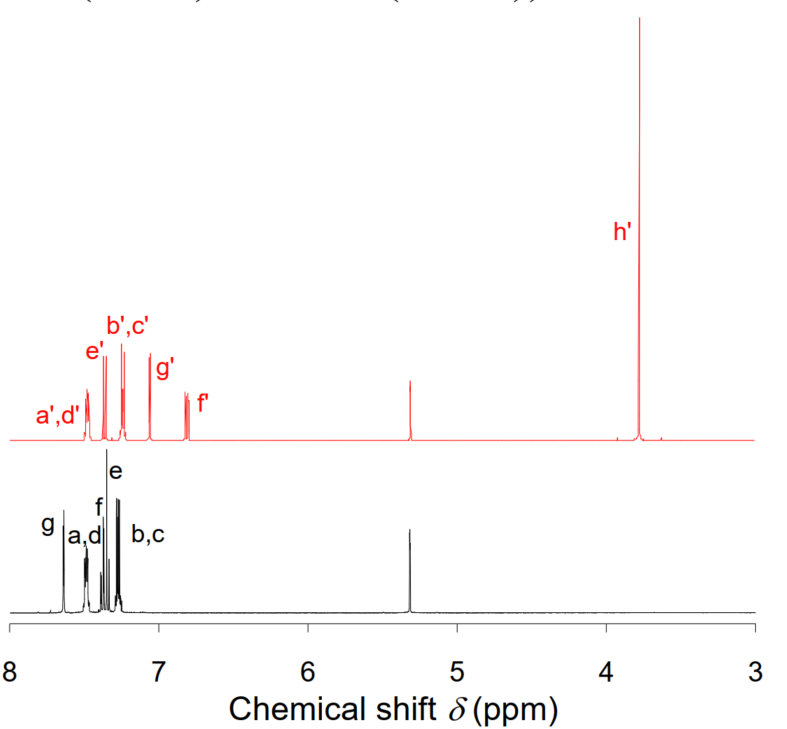


**Figure S1.** ^1^H-NMR spectra of BrTh (black) and MeO-Th (red) in CD_2_Cl_2_.

Scheme S2 Synthesis of CO-Th.


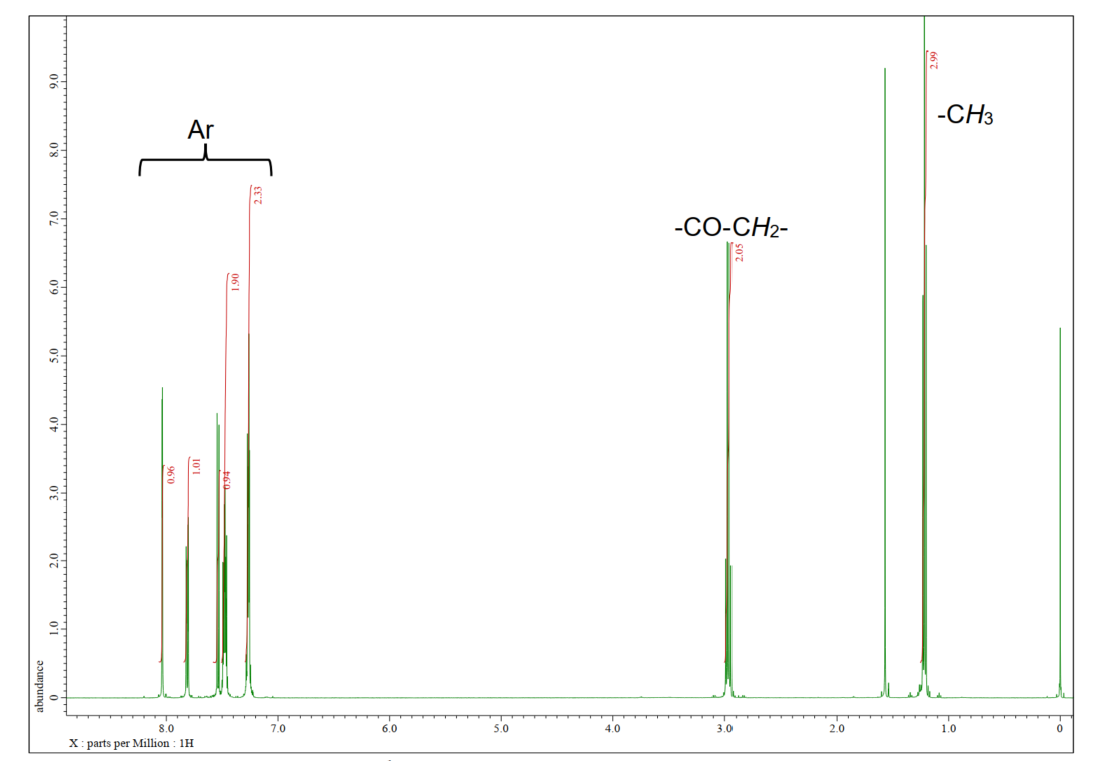


**Figure S2.** ^1^H-NMR spectrum of CO-Th in CDCl_3_.


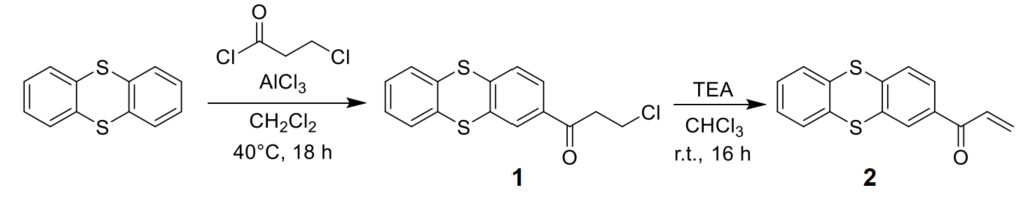

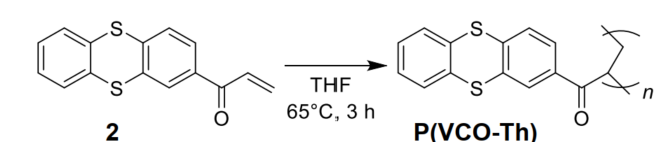


Scheme S3. Synthesis of P(VCO-Th).


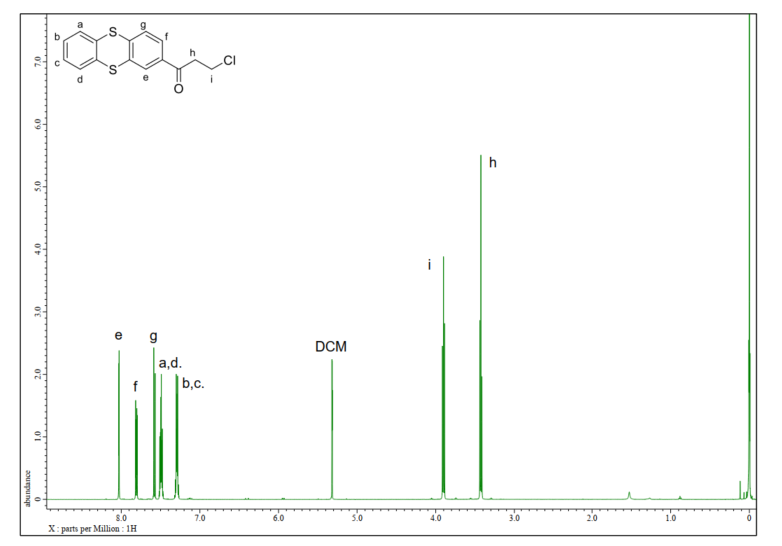


a)


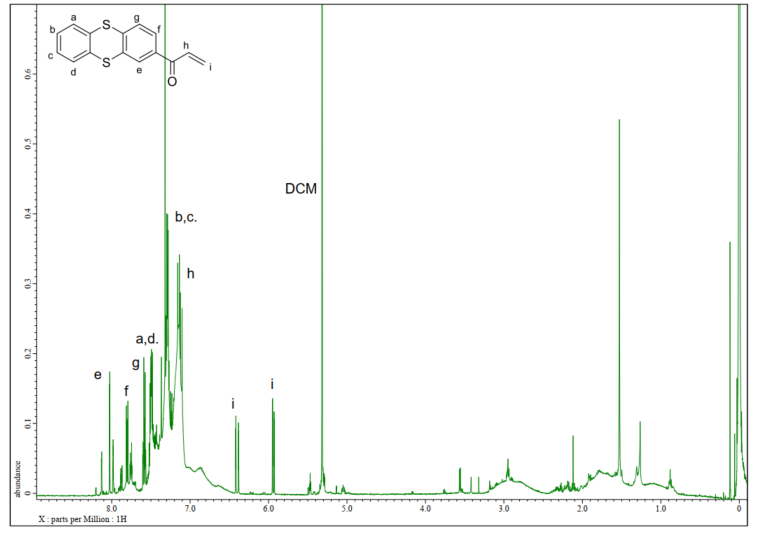


b)


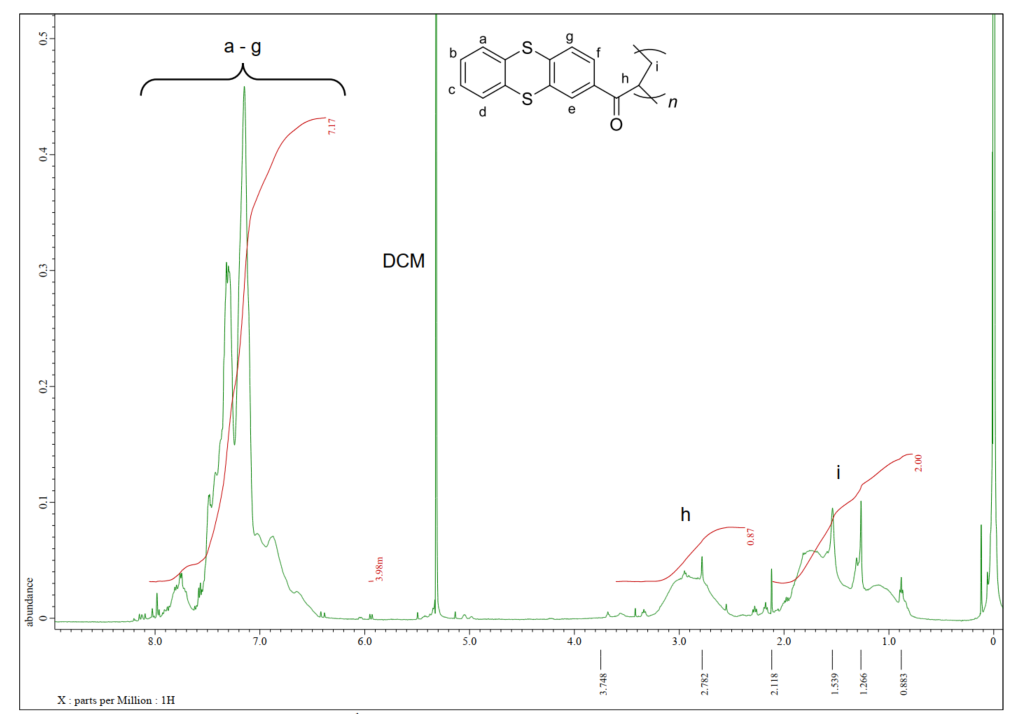


c)

**Figure S3.** 1H-NMR spectra of a) **1**, b) **2**, and c) P(VCO-Th) in CD_2_Cl_2_. A noisy spectrum was observed with **2** because it was highly reactive and difficult to be purified by recrystallization.


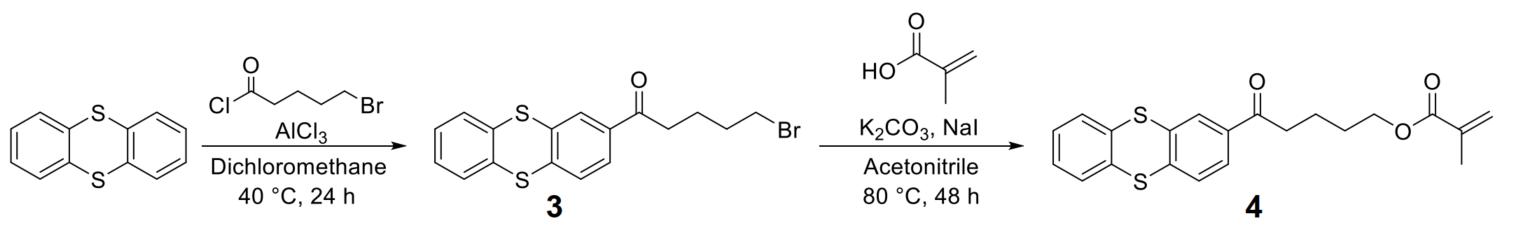


Scheme S4. Synthesis of 3 and 4.


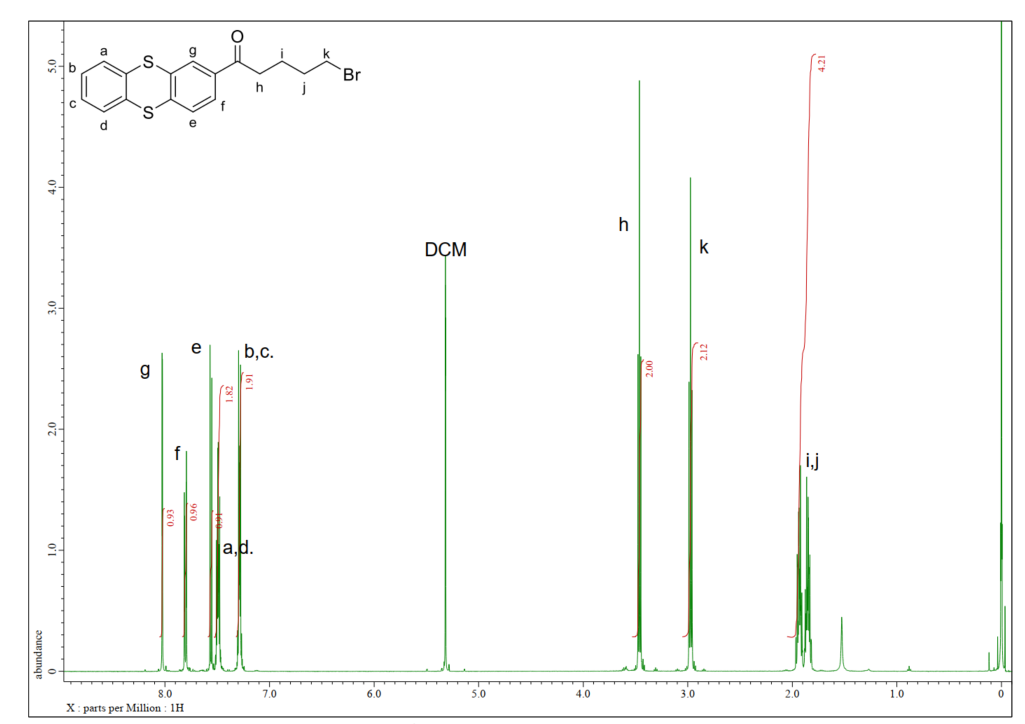


a)


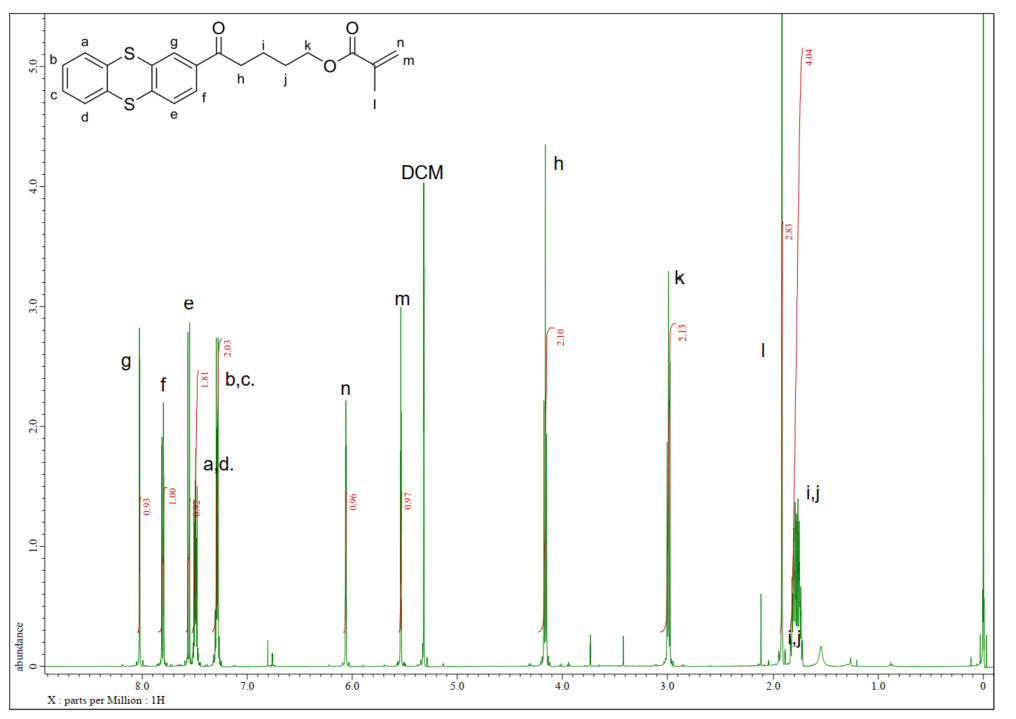


b)

**Figure S4.** ^1^H-NMR spectra **3** and **4** in CD_2_Cl_2_.

Scheme S5. Synthesis of P(BtCO-Th).


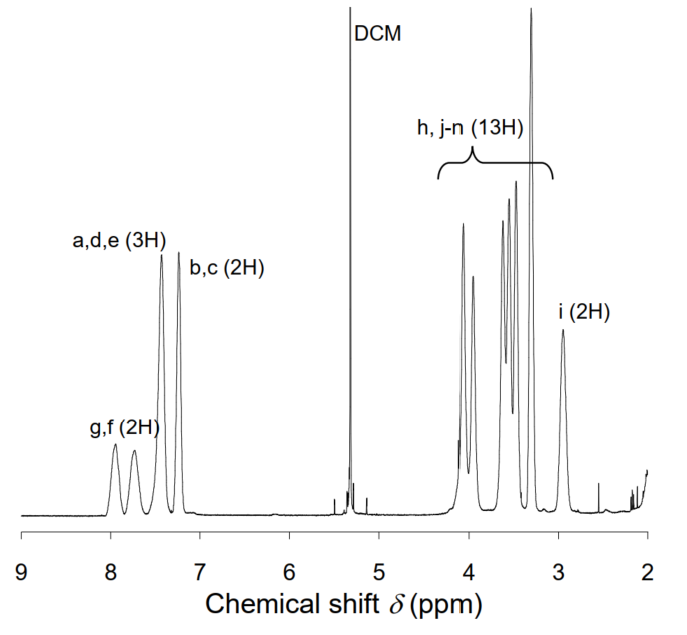


**Figure S5.** ^1^H-NMR spectrum of P(BtCO-Th) in CD_2_Cl_2_ (Run2).

Table S1. Synthesis of P(BtCO-Th) with different conditions.

Scheme S6. Synthesis of PTMA.


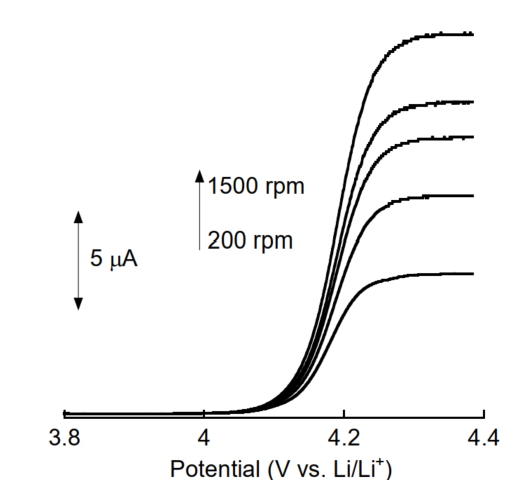

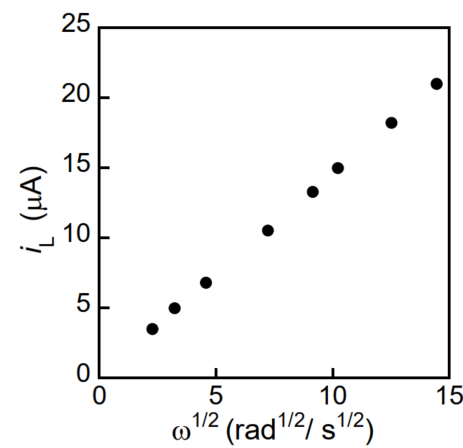


a) b)


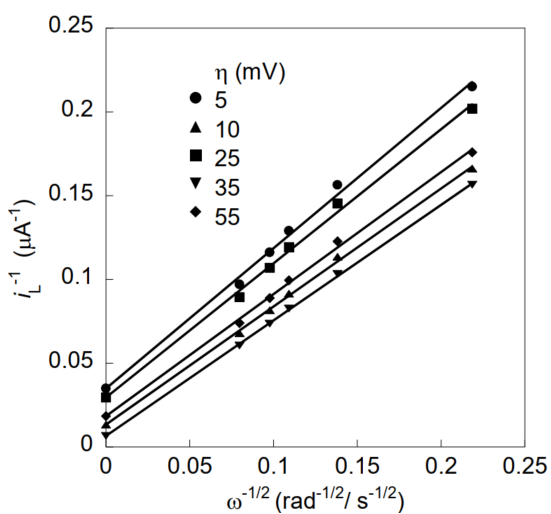

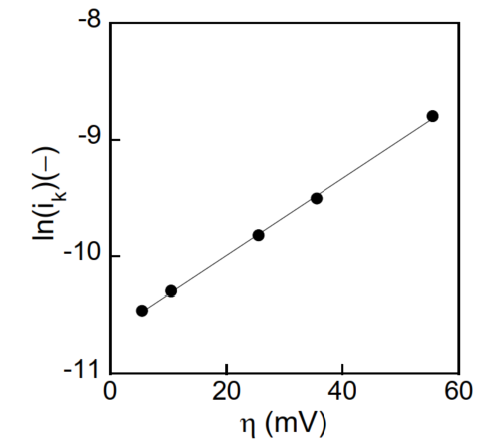


c) d)

**Figure S6.** RDE measurement results for the 1 mM CO-Th solution. a) Hydrodynamic voltammograms scanned at 10 mV/s. b) Levich plot. c) Koutecký-Levich plot. d) Tafel plot.


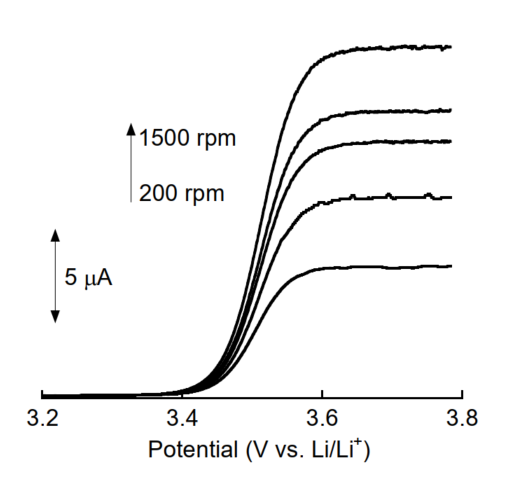

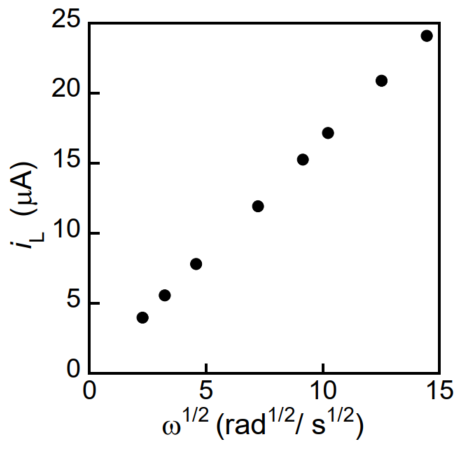


a) b)


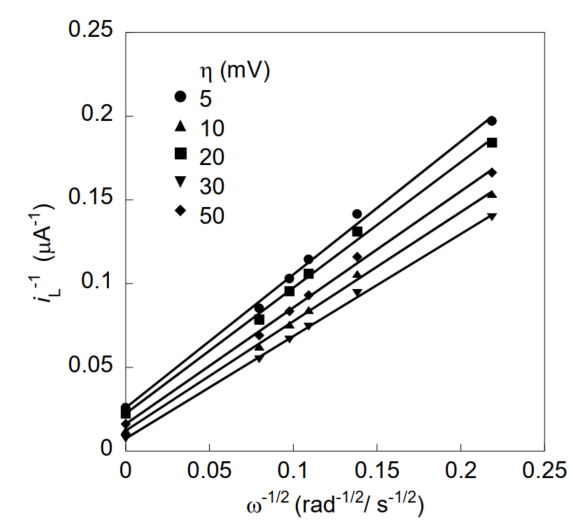

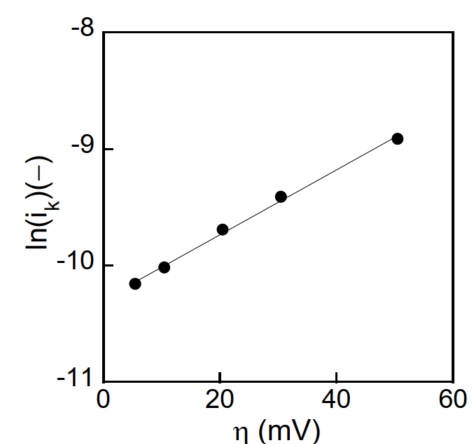


c) d)

**Figure S7.** RDE measurement results for the 1 mM TEMPO solution. a) Hydrodynamic voltammograms scanned at 10 mV/s. b) Levich plot. c) Koutecký-Levich plot. d) Tafel plot.

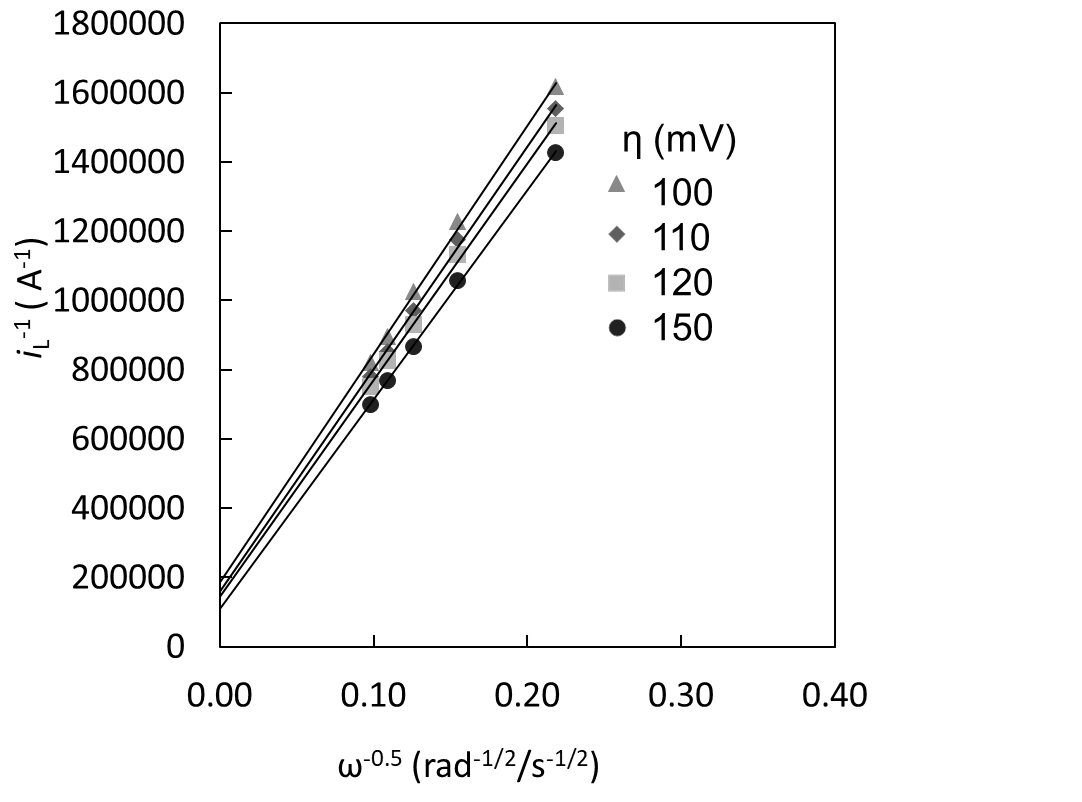

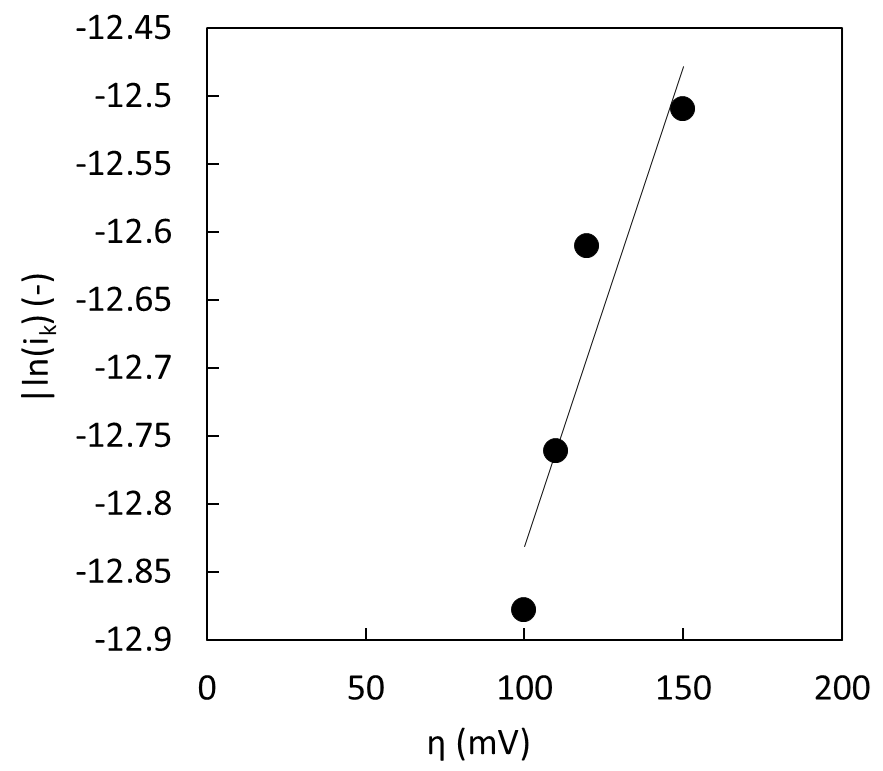


**Figure S8.** RDE measurement results for the 2 mM P(VCO-Th) dispersion. a) Hydrodynamic voltammograms scanned at 10 mV/s. b) Levich plot. c) Koutecký-Levich plot. d) Tafel plot.

a) b)


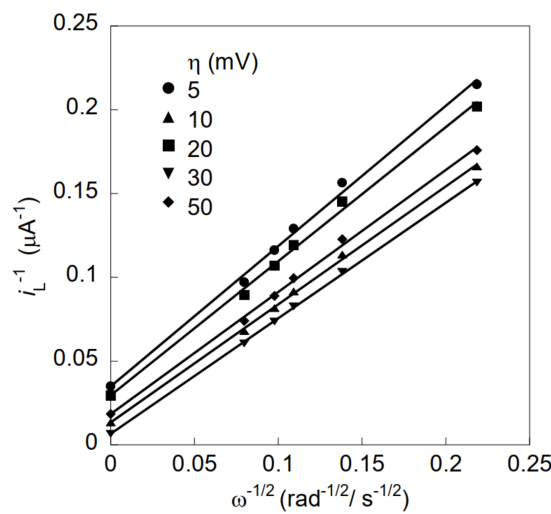

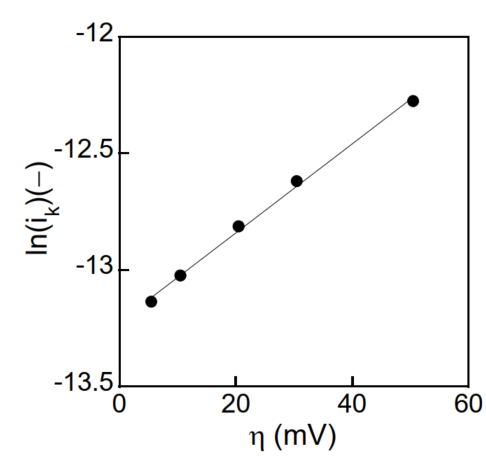


c) d)

**Figure S9.** RDE measurement results for the 1 mM P(BtCO-Th) solution. a) Hydrodynamic voltammograms scanned at 10 mV/s. b) Levich plot. c) Koutecký-Levich plot. d) Tafel plot.


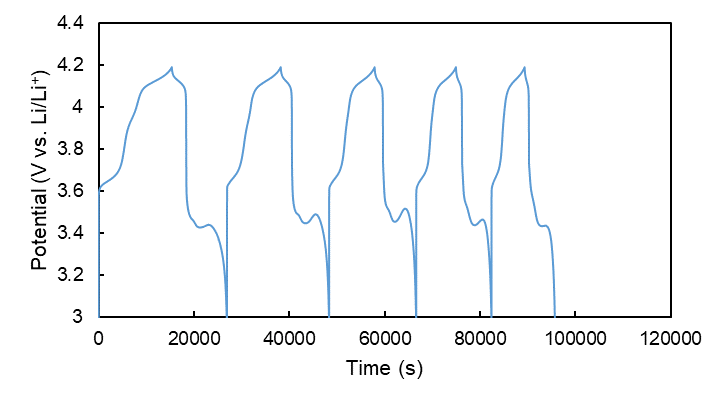


a)


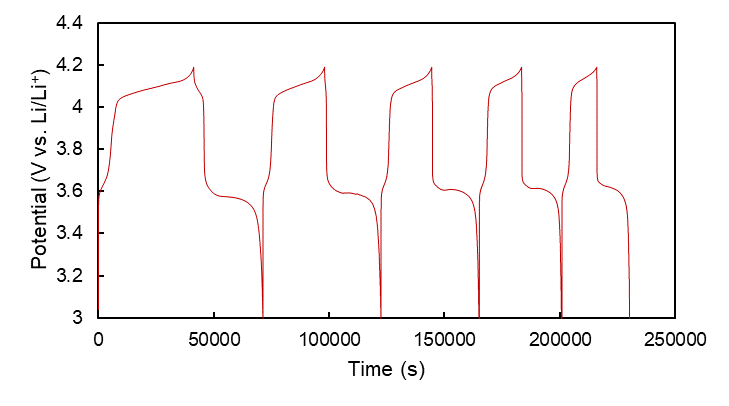


b)


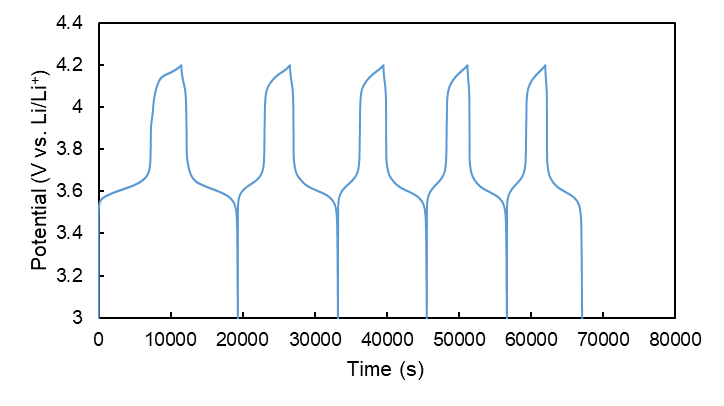


c)


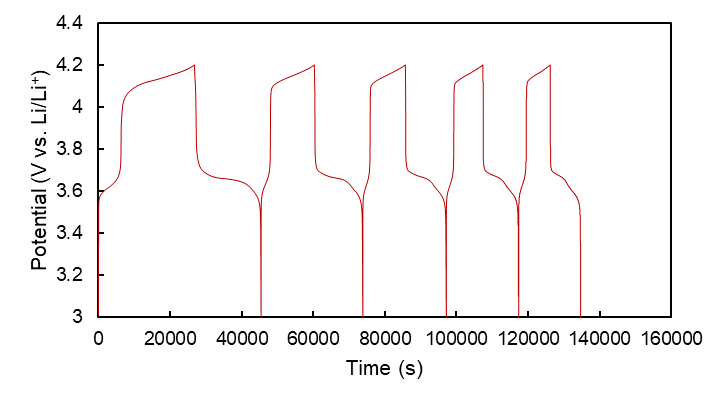


d)

**Figure S10.** Cycle performances of the polymer-based cells. a) Entry 5, b) 6, c) 7, and d) 8. Charging and discharging were repeated five times at 0.25 C for mediators. The initial curves are also shown in Figure 4a.
